# How New Mexico Leveraged a COVID-19 Case Forecasting Model to Preemptively Address the Health Care Needs of the State: Quantitative Analysis

**DOI:** 10.2196/27888

**Published:** 2021-06-09

**Authors:** Lauren A Castro, Courtney D Shelley, Dave Osthus, Isaac Michaud, Jason Mitchell, Carrie A Manore, Sara Y Del Valle

**Affiliations:** 1 Information Systems & Modeling Group Analytics, Intelligence and Technology Division Los Alamos National Laboratory Los Alamos, NM United States; 2 Center for Nonlinear Studies Los Alamos National Laboratory Los Alamos, NM United States; 3 Statistical Sciences Group Computer, Computational, and Statistical Sciences Division Los Alamos National Laboratory Los Alamos, NM United States; 4 Presbyterian Health Services Albuquerque, NM United States

**Keywords:** COVID-19, forecasting, health care, prediction, forecast, model, quantitative, hospital, ICU, ventilator, intensive care unit, probability, trend, plan

## Abstract

**Background:**

Prior to the COVID-19 pandemic, US hospitals relied on static projections of future trends for long-term planning and were only beginning to consider forecasting methods for short-term planning of staffing and other resources. With the overwhelming burden imposed by COVID-19 on the health care system, an emergent need exists to accurately forecast hospitalization needs within an actionable timeframe.

**Objective:**

Our goal was to leverage an existing COVID-19 case and death forecasting tool to generate the expected number of concurrent hospitalizations, occupied intensive care unit (ICU) beds, and in-use ventilators 1 day to 4 weeks in the future for New Mexico and each of its five health regions.

**Methods:**

We developed a probabilistic model that took as input the number of new COVID-19 cases for New Mexico from Los Alamos National Laboratory’s COVID-19 Forecasts Using Fast Evaluations and Estimation tool, and we used the model to estimate the number of new daily hospital admissions 4 weeks into the future based on current statewide hospitalization rates. The model estimated the number of new admissions that would require an ICU bed or use of a ventilator and then projected the individual lengths of hospital stays based on the resource need. By tracking the lengths of stay through time, we captured the projected simultaneous need for inpatient beds, ICU beds, and ventilators. We used a postprocessing method to adjust the forecasts based on the differences between prior forecasts and the subsequent observed data. Thus, we ensured that our forecasts could reflect a dynamically changing situation on the ground.

**Results:**

Forecasts made between September 1 and December 9, 2020, showed variable accuracy across time, health care resource needs, and forecast horizon. Forecasts made in October, when new COVID-19 cases were steadily increasing, had an average accuracy error of 20.0%, while the error in forecasts made in September, a month with low COVID-19 activity, was 39.7%. Across health care use categories, state-level forecasts were more accurate than those at the regional level. Although the accuracy declined as the forecast was projected further into the future, the stated uncertainty of the prediction improved. Forecasts were within 5% of their stated uncertainty at the 50% and 90% prediction intervals at the 3- to 4-week forecast horizon for state-level inpatient and ICU needs. However, uncertainty intervals were too narrow for forecasts of state-level ventilator need and all regional health care resource needs.

**Conclusions:**

Real-time forecasting of the burden imposed by a spreading infectious disease is a crucial component of decision support during a public health emergency. Our proposed methodology demonstrated utility in providing near-term forecasts, particularly at the state level. This tool can aid other stakeholders as they face COVID-19 population impacts now and in the future.

## Introduction

Since the novel coronavirus SARS-CoV-2 was identified and declared a global pandemic on March 11, 2020 [1], a key concern has been whether the demand for health care will exceed available resources. Early case reports clearly demonstrated that a large number of infections resulted in hospitalization, intensive care unit (ICU) admission, and breathing assistance via mechanical ventilation [2]. Further projection studies, which show plausible outcomes under defined scenarios [3], showed that COVID-19, the disease caused by SARS-CoV-2, had the potential to overwhelm existing capacity, especially given its limited treatment options [4-7]. In areas with limited resources and high rates of transmission, health care capacity has indeed been exceeded [8]. This threat has highlighted the need for continuous monitoring of hospital resources and for forecasting the impact of real-time changes in new cases on future strain of the health care system.

Real-time forecasting of infectious diseases has become a crucial component of decision support during public health emergencies. Since 2013, when the US Centers for Disease Control and Prevention (CDC) began hosting an annual influenza forecasting challenge [9], the field of infectious disease forecasting has grown. In the context of influenza, the task is for modelers to supply probabilistic forecasts of influenza-like illness for short-term targets, such as week-ahead incidence, at multiple geographical scales, using a variety of models and methods. From this effort, forecasting attempts for other diseases, such as chikungunya [10], Ebola [11], and West Nile [12], have proliferated in recent years, laying the groundwork for a rapid pivot to forecasting COVID-19 incident cases, deaths, and hospitalizations [13].

However, in the context of predicting impact on the health care system, the stress resulting from COVID-19 cases depends not only on the number of new hospitalized individuals but also on their overlapping periods of hospitalization. To the best of our knowledge, health care use forecasting in a probabilistic sense had not existed prior to the current COVID-19 pandemic. Hospitals had used historical data to make time series– and regression-based projections for long-term planning (ie, planning for the next 1 to 10 years). Generally, these projections examined a single relevant metric, such as average length of stay (ALOS) [14], discharges [15], demand for specific hospital specialties [16], or occupancy/bed need [17,18], and considered the impact of external trends, such as anticipated sociodemographic changes, through consideration of multiple scenarios [18]. Hospitals were also developing short-term forecasting tools of total occupancy or bed use [19-21], total occupancy as predicted by emergency department visits [22], and various emergency department metrics [23-26]. These short-term prediction efforts tended to approach the problem either from a hospital administration perspective, by focusing on operational measures informed by a single hospital’s [19-22] or department’s [23-26] historic data or surgery schedule [27], or from a research perspective by using hospital time series data as a use case for the development and assessment of novel statistical models [28-31] without regard to hospitals as complex systems in response to a burgeoning pandemic.

In response to the overwhelming demand from government agencies at the federal, state, and local levels to predict the immediate future burden of COVID-19, Los Alamos National Laboratory (LANL) first developed the COVID-19 Forecasts Using Fast Evaluations and Estimation (COFFEE) tool [32]. COFFEE generates short-term (1 day to 6 weeks ahead) forecasts of cumulative and incident confirmed cases and deaths for all US counties and states, as well as all countries with at least 100 confirmed cases and 20 deaths as reported by the Center of Systems Science and Engineering (CSSE) at the Johns Hopkins University (JHU) Coronavirus Resource Center dashboard [33]. To forecast the health care needs expected to arise from predicted cases, we additionally created the COVID-19 Hospitalization, Intensive Care, and Ventilator Estimator (CHIVE) forecasting tool, which combines forecasts every Monday and Thursday from COFFEE for the state of New Mexico, with current state-level data on hospitalizations, ICU bed use, and mechanical ventilator use. CHIVE is most useful as an actionable mid-term (ie, 2 to 4 weeks ahead) predictor of hospital resource use, enabling hospitals to order additional supplies and allocate existing staff and resources to best serve an expected influx of patients.

The aim of this modeling effort was to create a flexible forecasting tool to predict COVID-19–related health care use needs 2 to 4 weeks ahead, a time period identified by state and local stakeholders as being most useful for future planning. Here, we present the CHIVE methodology and characterize its performance over 29 forecasts made between September 1 and December 9, 2020, for New Mexico. This performance period includes both retrospective forecasts, as in, what the forecast would have been if the data were available, and those submitted in real time to the New Mexico Department of Health (NMDOH). The results of this study can provide a platform for other research groups or health departments to estimate health care needs and support decisions regarding resource planning and allocation.

## Methods

### Data

**Figure 1:**
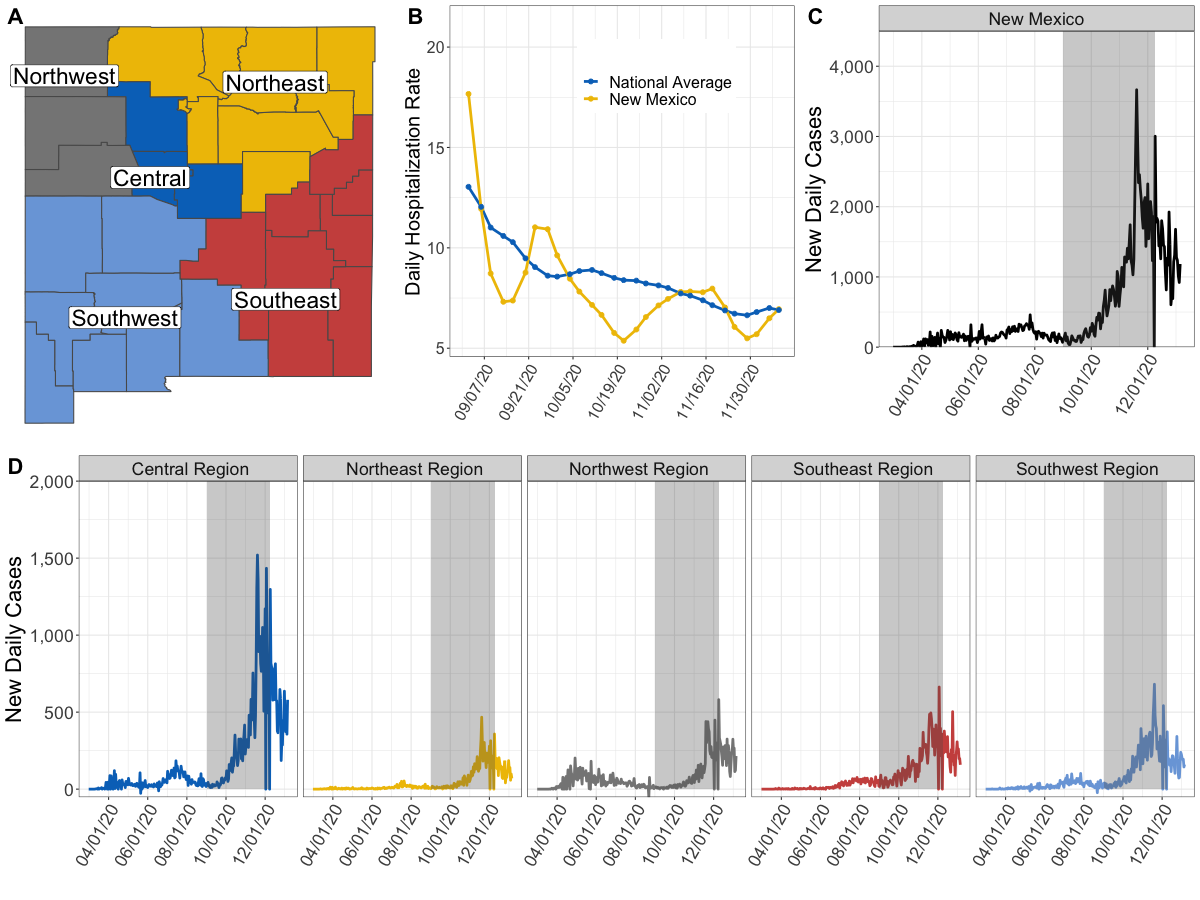
New Mexico health regions, COVID-19 confirmed case time series from March 2020 to January 2021, and daily hospitalization rates from September to December 2020. (A) Division of New Mexico’s 33 counties into five health regions. (B) The 2-week weighted daily hospitalization rate for New Mexico (yellow) compared to the national average (blue) (C). The number of new daily cases at the state level, with the grey box denoting the period of forecast performance evaluation. (D) The number of new daily COVID-19 cases for each of the five regions.

From November 4, 2020, to January 5, 2021, as a result of our partnership with NMDOH and Presbyterian Healthcare Services, we received access to the statewide number of COVID-19 confirmed/suspected inpatient hospitalizations, the number of patients in ICUs, and the number of patients on mechanical ventilation as reported in EMResource, a web-based tool for hospital resource reporting [34]. This data set contained retrospective data beginning from July 20, 2020. We also received individual hospital data on COVID-19 hospitalizations. For each day of data, we created regional level time series by summing the numbers of confirmed COVID-19 inpatient hospitalizations, patients in ICUs, and patients on mechanical ventilation across the hospitals within the counties assigned to each of NMDOH’s five health regions (Figure 1A, Figure S1 in Multimedia Appendix 1).

### Daily Hospitalization Rate

Starting November 3, 2020, the United States Department of Health and Human Services began publishing a time series format of daily numbers of new hospitalizations by state [35], hereafter referred to as the HealthData.gov time series, with retrospective data for New Mexico going back to July 14, 2020. These data were released weekly.

We defined the daily hospitalization rate (DHR) as the ratio of new confirmed COVID-19 hospitalizations to new confirmed COVID-19 cases on the same day. To obtain the number of new confirmed COVID-19 hospitalizations from the HealthData.gov time series, we summed the previous day’s admissions of adults and pediatric COVID-19 cases. We used the number of new cases as reported by the CSSE JHU Coronavirus Resource Dashboard [33] as the number of new confirmed individuals with COVID-19. We aligned the HealthData.gov admission data for day *t* with the JHU data of *t* – 1 because the admission data are listed as the previous day. To account for data reporting anomalies (eg, “data dumps,” days with zero reported hospitalizations or new cases), we calculated a 7-day rolling average of each quantity. The DHR on day *t,* therefore, is the 7-day rolling average of hospitalizations divided by the 7-day rolling average of new COVID-19 cases. Because the HealthData.gov time series only provides state-level numbers, we applied the same *DHR*(*t*) to the regional forecasts.

The daily number of new hospitalizations in the future will impact the degree to which overlapping lengths of stay strain hospital resource capacity. To forecast the DHR into the future, *DHR*(*t*)*'*, we calculated a 2-week time-weighted average of the DHR and then assumed this DHR would persist throughout the forecast duration. Where *t*= 0 is the last day of the observed data, the weight of each *DHR* (*t* – *n*) for *n* = 0: 13 was determined as


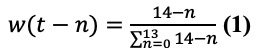


The DHR is a convenient ratio that is obtainable from available data. However, this metric is incorrectly defined because the denominator population is not included in the numerator population; thus, we used a 2-step modeling process to remove this inherent bias. Our goal was to create the most accurate forecast possible and not necessarily to maintain direct interpretation of the variables used in the model. A more traditional metric, such as case hospitalization rate, would require further inference about the lag between new cases and hospitalizations of those same cases, which is not necessary to achieve an accurate forecast.

### Baseline Parameters of Health Care Use

The stress that COVID-19 places on New Mexico’s health care capacity depends not only on the rate of new hospitalizations but also on the amount of overlapping time during which individuals are hospitalized, which itself is dependent on the individual illness severity and length of stay for each patient. Therefore, we structured CHIVE around the probabilities that certain hospital events would occur (ie, ICU need) given hospitalization and the length of hospitalization given illness severity. Specifically, our model depended on four parameters: ALOS, ALOS for those admitted to the ICU or requiring mechanical ventilation, and percentages of those hospitalized and later admitted to the ICU and those hospitalized who were later placed on mechanical ventilation (Table 1). Early evidence showed that these parameters varied across age groups, and later evidence showed that they varied temporally as new treatment protocols were established [36]. For example, early in the pandemic, individuals were frequently placed on ventilators, leading to high percentages of hospitalized individuals on mechanical ventilation. In addition, it is uncertain how spatial variation in underlying health conditions and transmission intensity impact health care patterns.

Baseline parameters were estimated from data on New Mexico’s hospitalized COVID-19 cases from April 16 to June 15, 2020 [37]. In this time period, the ICU percentage of hospitalizations ranged from 37% to 53% and the percentage of patients on ventilation ranged from 22% to 36%. Rather than dynamically inferring the parameters with limited noisy data going forward, we used parameters that reflected these initial health care use trends in New Mexico, and we developed a postprocessing procedure (described in *Step 2: Postprocessing Based on Back-Fitting*) that adjusted the forecasts to time-varying trends without the need to determine new input values.

**Table 1:** New Mexico baseline parameters for COVID-19 health care use needs based on data from March to June 2020.

Parameter	New Mexico baseline	US range^a^
Average length of inpatient stay, days	5	4-6
Average length of stay for those admitted to the ICU^b^ or on mechanical ventilator, days	14	11-14
Patients admitted to ICU among those hospitalized, %	42.8	23.8-36.1
Patients on mechanical ventilation among those hospitalized, %	28.2	12.0-22.1

^a^For comparison, we provide the best median estimate across age groups in the United States collected by the US Centers for Disease Control through August 8, 2020 [36].

^b^ICU: intensive care unit.

### CHIVE Forecasting Model

To capture the heterogeneity in the severity of individual infections, we used probabilistic simulation. CHIVE works in two steps. In the first step, 4-week-ahead daily forecasts, *θ_t_*_=1:28_, of new confirmed cases from COFFEE [32] were used as input data to simulate 4 weeks of health care use. We simulated health care use based on the forecasted number of new cases, *θ_t_*_=1:28_, the weighted two-week average *DHR*(*t*)*'*, and the baseline parameters. The output of the simulation is a forecast for the numbers of occupied inpatient beds (*H*_1_,..., *H*_28_), ICU beds (*IC*_1_,..., *IC*_28_) and in-use ventilators (*V*_1_,..., *V*_28_) due to COVID-19. After simulating 1000 independent iterations of *H_t_*_=1:28_, *IC_t_*_=1:28_, and *V_t_*_=1:28_, the second step adjusts the magnitudes of summary quantiles *q* of *H_t_*_=1:28_, *IC_t_*_=1:28_, and *V_t_*_=1:28_, based on observed differences between the prior weeks’ baseline forecasts and the subsequently observed data.

#### Step 1: Model Baseline Simulations

For an independent iteration *i* of the simulator, we first sampled a trajectory of new COVID-19 cases, *θ_t_*_=1:28_, from the distribution specified by the 23 quantiles of *θ_t_*_=1:28_ in the COFFEE output. Let *p*_i_~*Uniform*(0,1) be the percentile of *θ_t_*_=1:28_ sampled. We drew *θ_t_*_=1:28_ such that *p_i_* was the same for all *t* within iteration *i*.

For a day-ahead forecast *t* + *n* where *t*= 0 is the last day of observed data, we generated our forecasts as follows:

1. Using a binomial distribution with the success probability equal to *DHR(t)'*, we sampled the number of new hospital admissions *y_t+n,i_* using the forecasted number of new confirmed cases *θ_t+n,i_* on day *t+n* as the number of trials.
2. We next sampled the number of new individuals admitted to the ICU, *u_t+n,i_*, from a binomial distribution with *y_t+n,i_* trials and success probability equal to the ICU admission percentage among those hospitalized. Similarly, we sampled the number of new individuals needing mechanical ventilation *w_t+n,i_* from a binomial distribution with *y_t+n,i_* trials and probability equal to the percent of all hospital admissions that require mechanical ventilation. We assumed that if *w_t+n,i_*≤*u_t+n,i_*, all individuals requiring mechanical ventilation were also in an ICU; otherwise, we assumed that some non-ICU admissions also required mechanical ventilation. Thus, we calculated the number of new non-ICU or ventilator admissions (ie, general inpatient bed admissions), as *y'_t+n,i_ = y_t+n,i_* – max (*u_t+n,i_, w_t+n,i_*).
3. We next simulated the lengths of stay *S* for each new admission *z*. For admissions in an inpatient bed, we drew the lengths of stay from a Poisson distribution such that 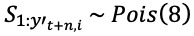.
4. Because we assumed that the length of stay was similar between ICU patients and those on mechanical ventilation, we drew the lengths of stay for these critical care individuals from a Poisson distribution where 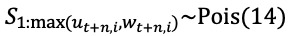. To obtain the lengths of stay for individuals in the group min (*u_t+n,i_*, *w_t+n,i_*), we sampled a subset without replacement from 
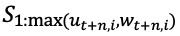
.
5. For all admissions observed up through *t+n*, we decreased the remaining length of stay for each individual by 1. The number of needed inpatient beds *H_t+n,i_* on *t+n* was then the number of individuals who have a positive length of stay remaining. Similarly, we tracked *IC_t+n,i_* and *V_t+n,i_*.

We repeated this process for 1000 random samples of *θ_t_*_=1:28_ and summarized the forecasts for day *t* + *n* by a set of 23 quantiles *q* at levels 0.01, 0.025, 0.05, 0.10, … , 0.95, 0.975, and 0.99, such that we obtained *H*_t=1:28,_*_q_*_=1:23_, *IC*_t=1:28,_*_q_*_=1:23_ and *V*_t=1:28,_*_q_*_=1:23_.

This method makes several simplifying assumptions. First, it assumes instantaneous movement from confirmation of COVID-19 to hospitalization. Second, it assumes that a hospitalized individual requires the same category of health care for the duration of their stay. We argue that because the forecasting model is not meant to infer epidemiologic parameters, these simplifications reduce the need for introducing additional parameters when data may not exist to sufficiently estimate them.

#### Step 2: Postprocessing Based on Back-Fitting

After generating the baseline 4-week forecasts *H*_t=1:28,_*_q_*_=1:23_, *IC*_t=1:28,_*_q_*_=1:23_, and *V*_t=1:28,_*_q_*_=1:23_, we adjust their magnitudes by finding scaling factors that bring the past week’s baseline forecasts into alignment with the observed data (Table S1, Multimedia Appendix 1). In this way, we do not need to adjust the baseline parameters.

**Figure 2:**
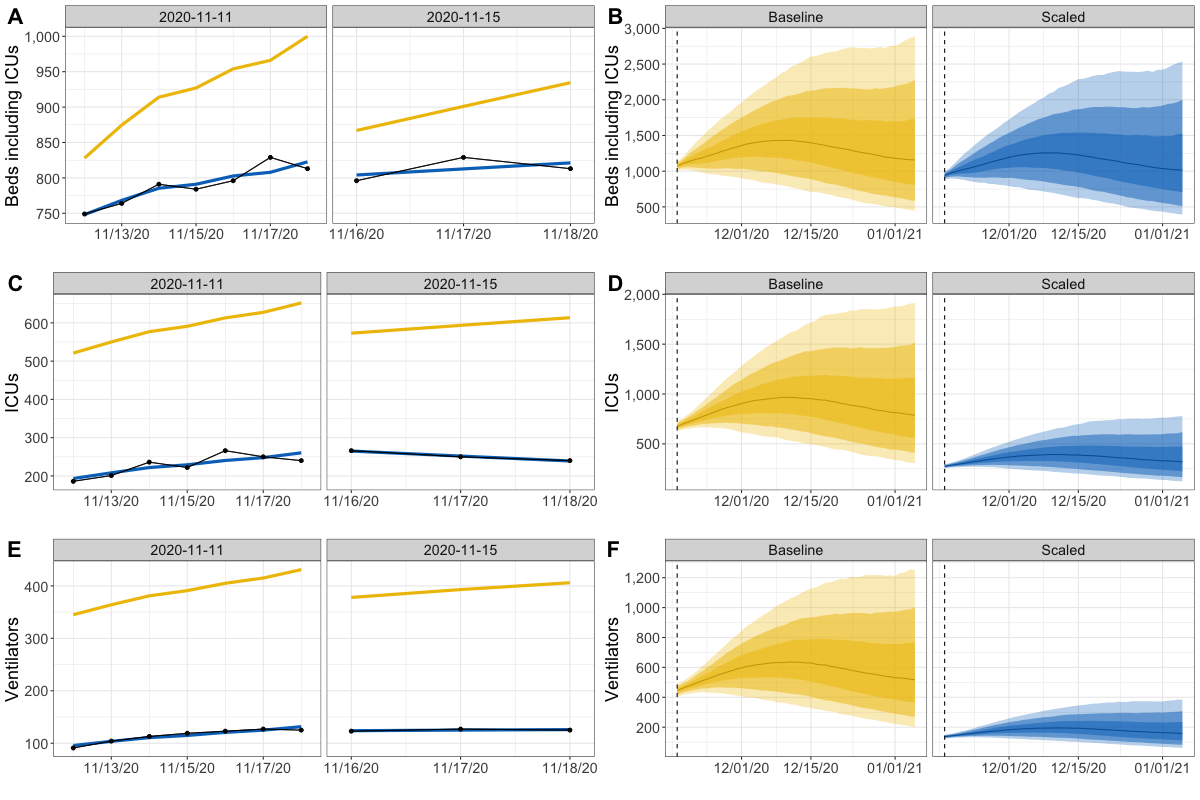
Back-fitting procedure for the November 18, 2020, forecast for New Mexico. (A, C, E) For each forecast from over the week (the panels), a regression was fit (blue line) from the 50th percentile of the baseline forecasts (yellow line) to the observed data (black dots). A time-weighted average of the 2 regression coefficients from each panel was calculated separately for inpatient beds (A), ICU beds (C), and ventilators (E). (B, D, F) For the November 18 forecast, the *Baseline* forecast was multiplied by the time-weighted average of the regression coefficients to produce the *Scaled* forecast for the next four weeks. ICU: intensive care unit.

We fitted linear regression models from the forecasts generated over the past week to the observed data as follows for variable *X*, where *X* is either *H*, *IC*, or *V* (Figure 2):

1. Let *t*=0 be the day of the last observed data. For each forecast *X_t–n_*, *n*=1,..., 7, we fit a linear regression model from the 50th percentile *X_t–n, q=50_* baseline trajectory to the eventually observed data *Y_t–n:t_*, in the form *Y_t–n_* = *β_t–n_X_t–n,q=50_*.
2. Across the forecasts, we take a time-weighted mean of the regression coefficients *β_t–n:t_*, assigning weights as in Equation 1.
3. We multiply all baseline forecast quantiles by the weighted mean 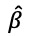 to obtain *X'_t=1:28,q=1:23_*.

We found 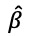 separately for inpatient beds, ICU beds, and in-use ventilators. All simulations and analyses were conducted using R, version 3.6.1 (R Foundation for Statistical Computing) [38].

## Results

### Back-Fit Coefficients Are Dynamic by Time and Geography

**Figure 3:**
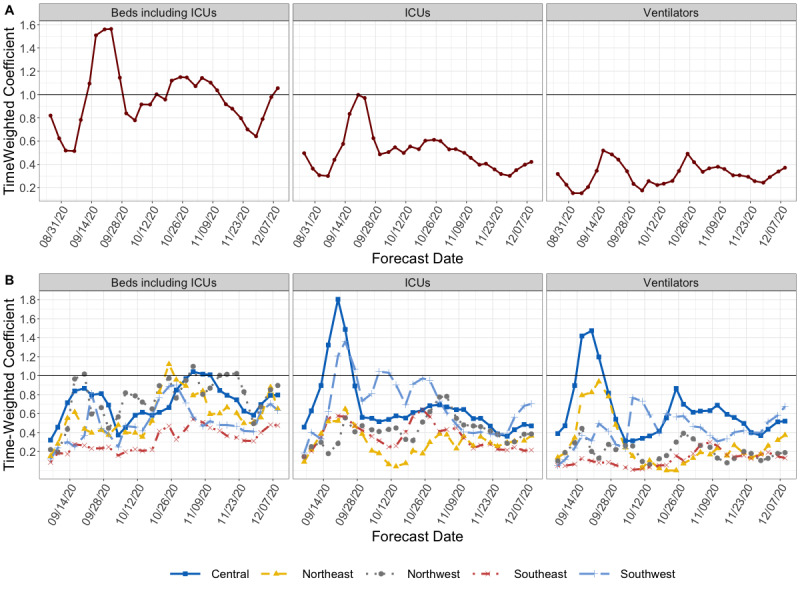
The fitted scaling coefficients from September to December, 2020, for hospital inpatient beds, ICU beds, and ventilators at the state level (A) and regional level (B). The solid black line represents a coefficient of 1.0, where the original 50th percentile forecast would be a good match for the eventual observed data. ICU: intensive care unit.

At the state level, the 1-week weighted coefficients 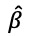 fluctuated through time (Figure 3A). All three health care use coefficients showed three peaks: one in early September, a second in late October, and a third in early December. During the first two peaks, the baseline forecasted number of inpatient beds was underestimated (coefficients greater than 1.0), while the baseline forecasts of ICU beds and individuals on ventilators were consistently overestimated (coefficients less than 1.0).

At the regional level, the baseline parameters most often produced forecasts that were overestimates for each of the five New Mexico regions (Figure 3B). In contrast to the state level, baseline forecasts of needed ICU beds and ventilators were underestimated for the Central region during mid-September. Across health care use categories, the coefficients for the Central region were closest to 1.0, indicating that the baseline parameters were the best match for this region. The coefficients for the Southeast region were consistently the smallest, indicating that the baseline parameters did not reflect health care trends in this region.

### Forecasts Showed Higher Error at the Regional Level and for Ventilators

**Figure 4:**
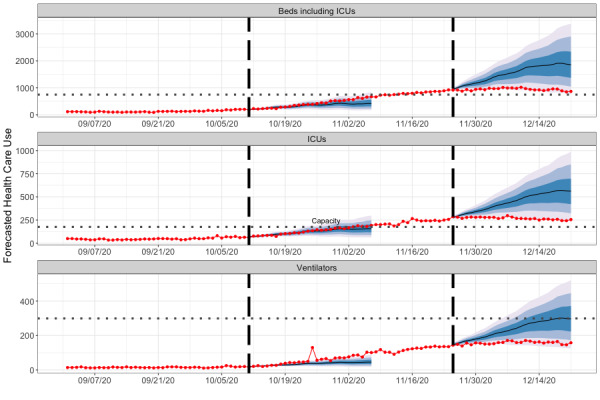
Reported health care use and example forecasts for New Mexico from October and November 2020. The numbers of concurrent hospitalization beds, ICU beds, and ventilators needed throughout hospitals in New Mexico from September 1 to December 29, 2020 (red points and line). Forecasts are day-ahead predicted medians (black line), and the 50%, 80% and 95% prediction intervals for 28 days (4 weeks). We show two examples, the first beginning on October 11, 2020, and the second beginning on November 25, 2020. ICU: intensive care unit.

Using validation data through January 5, 2021, we compared the accuracy of the 4-week forecast horizons for the 29 forecasts made between September 1 and December 9, 2020 (Figure 4). To compare accuracy across health care use categories, regions, and time—where the observed magnitude varies widely—we looked at the weighted absolute percentage error (WAPE) while providing the mean absolute error (MAE) for context. The MAE is the difference between the median forecast (50th percentile) and the observed value. The WAPE is the sum of the absolute differences divided by the sum of the observed values over the 4-week forecast horizon. The WAPE can accommodate observed zero values, which occurred in our data at the regional level.

At the state level, the median forecasts were consistently 15% to 25% off for all three health care use categories for the 1- to 2-week horizon, and they showed an increase in error up through 4 weeks ahead (Table 2). Looking at the completed months during which a forecast was made, the October forecasts had the lowest overall mean WAPE of 20.0%, while the September forecasts had the highest WAPE of 39.7%. Of the three health care use categories, inpatient bed forecasts had the lowest overall WAPE (27.3%), while ICU beds had the highest (29.2%).

The regional level WAPEs similarly increased though the 3-week forecast horizon; however, for each forecast horizon, the regional WAPEs often exceeded their corresponding state-level WAPEs. Aggregated across forecast horizons and regions, the regional-level WAPE was 40.0% for inpatient hospitalizations, 40.4% for ICU units, and 40.0% for ventilators. However, because of smaller quantities, these errors translate to smaller absolute errors. For example, in the Northwest region, ventilator median forecasts were off by between 55% and 75% on average, corresponding to a raw difference of approximately 3 ventilators.

At the regional level, forecast error varied by location, month, and health care use category. All three health care use categories in the Central region had the lowest forecast WAPEs in October (hospital beds: 33.7%, ICU beds: 25.0%, ventilators: 28.6%), while the lowest forecast WAPEs for the Southwest regions all occurred in November (hospital beds: 39.8%, ICU beds: 42.7%, ventilators: 35.3%). For the remaining combinations of regions and health care needs, the results were split, with 66% of the lowest WAPEs occurring in November.

To understand how time series properties may have impacted the accuracy of the forecasts, we compared the monthly WAPE against the monthly volatility of the time series. The volatility, calculated as 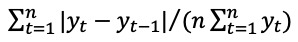, is a measure that captures the noisiness of the observed time series data. We found no relationship between the volatility of a monthly time series and the WAPE. We also did not find a relationship between the WAPE and the total number of each health care use need in a particular month.

**Table 2:** The WAPEs and MAEs of the predicted forecasted median for COVID-19 health care use numbers reported for September 1 to January 5, 2020, in New Mexico.

Region^a^ and type			Forecast horizon^b^													
			1 week ahead					2 weeks ahead				3 weeks ahead			4 weeks ahead	
			WAPE^c^		MAE^d^ (SD)		WAPE		MAE (SD)		WAPE		MAE (SD)	WAPE		MAE (SD)
**State**																
	Beds including ICUs^e^	0.170		81.8 (91.9)		0.244				130 (141)	0.307		179 (206)	0.334		210 (249)
	ICUs	0.163		23.6 (22.4)		0.244				38.8 (39.4)	0.338		58.3 (64.1)	0.380		69.6 (81.0)
	Ventilators	0.188		13.5 (13.6)		0.261				21.2 (21.9)	0.324		29.2 (31.4)	0.349		34.2 (40.3)
**Central**																
	Beds including ICUs	0.175		27.3 (33.9)		0.267				46.9 (60.9)	0.381		74.0 (97.9)	0.486		103 (113)
	ICUs	0.177		12.1 (13.7)		0.257				19.5 (23.4)	0.385		31.8 (39.7)	0.485		42.8 (50.6)
	Ventilators	0.204		9.43 (10.7)		0.281				14.7 (17.3)	0.358		20.9 (26.2)	0.406		25.9 (32.6)
**Northwest**																
	Beds including ICUs	0.283		12.8 (19.5)		0.335				17.9 (27.6)	0.425		26.5 (39.7)	0.480		34.0 (44.2)
	ICUs	0.261		3.62 (4.09)		0.368				5.81 (7.07)	0.482		8.82 (11.3)	0.516		10.6 (14.2)
	Ventilators	0.557		2.06 (2.17)		0.673				2.76 (2.80)	0.757		3.53 (4.30)	0.729		3.73 (5.44)
**Southeast**																
	Beds including ICUs	0.240		8.89 (7.59)		0.310				12.5 (12.9)	0.394		17.1 (16.4)	0.468		21.9 (19.5)
	ICUs	0.271		5.49 (4.55)		0.367				7.98 (7.64)	0.446		10.1 (10.2)	0.425		9.90 (10.1)
	Ventilators	0.346		2.17 (2.21)		0.468				3.34 (3.43)	0.547		4.21 (4.55)	0.605		5.03 (4.86)
**Southwest**																
	Beds including ICUs	0.251		15.1 (14.4)		0.413				26.9 (30.0)	0.595		41.0 (44.2)	0.618		45.0 (43.0)
	ICUs	0.249		10.1 (9.47)		0.410				18.2 (21.1)	0.562		26.6 (33.5)	0.601		30.0 (34.4)
	Ventilators	0.240		5.32 (5.04)		0.366				9.06 (9.40)	0.412		11.4 (10.6)	0.428		12.9 (9.95)
**Northeast**																
	Beds including ICUs	0.306		10.4 (13.3)		0.417				15.7 (20.1)	0.584		24.6 (30.4)	0.672		30.8 (34.8)
	ICUs	0.311		3.25 (3.64)		0.434				5.27 (6.16)	0.570		7.63 (9.51)	0.650		9.46 (10.3)
	Ventilators	0.408		2.21 (2.34)		0.514				3.33 (3.15)	0.582		4.20 (3.83)	0.609		4.69 (4.11)

^a^Regions are listed in increasing order of overall WAPE across both the forecast horizon and health care categories.

^b^For each forecast horizon, we considered all daily forecasts within that week.

^c^WAPE: weighted absolute percentage error.

^d^MAE: mean absolute error.

^e^ICUs: intensive care units.

### Prediction Intervals Start Off Narrow and Increase With Time

**Figure 5:**
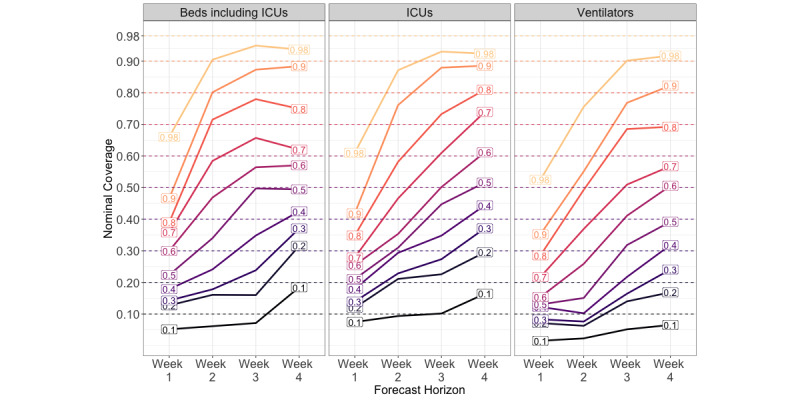
Coverage plot for New Mexico state-level forecasts at 4-week ahead horizons made between September 1 and December 9, 2020. Colored lines are labeled by their nominal coverage, while the position on the y-axis indicates its empirical coverage. If a forecast is well-calibrated, the empirical coverage should fall along the y-axis at its corresponding nominal coverage. ICU: intensive care unit.

We assessed how well the forecasts were calibrated by measuring how often the observed data fell within a range of prediction intervals. If a forecasting model is well calibrated (ie, the prediction intervals are the correct width), the observed data should fall into the nominal prediction interval of the model with the expected frequency. For example, the observed data should fall into the 50% nominal prediction interval 50% of the time. Across the two geographic regions, prediction intervals were conservative (overconfident) at both the 50% and 90% prediction intervals. At the state level, the empirical coverage approached the nominal coverage by the 3- and 4-week-ahead forecast horizons (Figure 5, Table 3) for inpatient beds and needed ICU beds. However, across the regional levels and health care categories, the prediction intervals remained consistently narrow.

At the state level, we ranked the calibrations by comparing the relative absolute coverage error, as in, (nominal coverage – observed coverage)/nominal coverage. Between health care categories, the hospitalization forecasts were the best calibrated at both geographic scales, while the ventilator forecasts were the worst calibrated. At the regional scale, the Central region was the best calibrated model, while the Northeast region was the worst (Figure S3, Multimedia Appendix 1).

**Table 3:** Observed prediction interval coverage for COVID-19 health care use needs in New Mexico reported from September 1 to December 9, 2020.

Model and type			Measure (% coverage)		Observed prediction interval coverage			
				Forecast horizon				
				1 week ahead		2 weeks ahead	3 weeks ahead	4 weeks ahead
**State**								
	Beds including ICUs^b^	50		0.22		0.34	*0.50* ^a^	*0.50*
		90		0.47		0.80	*0.87*	*0.88*
	ICU	50		0.21		0.31	*0.45*	*0.52*
		90		0.42		0.76	*0.88*	*0.89*
	Ventilators	50		0.13		0.15	0.32	0.39
		90		0.35		0.55	0.77	0.82
**Regional^c^**								
	Beds including ICU	50		0.27		0.33	0.36	0.30
		90		0.54		0.69	0.70	0.60
	ICU	50		0.29		0.29	0.36	0.38
		90		0.57		0.65	0.70	0.71
	Ventilators	50		0.20		0.23	0.27	0.33
		90		0.49		0.51	0.62	0.67

^a^Italicized quantities are within 5% of their nominal coverage.

^b^ICUs: intensive care units.

^c^Regional results are averaged across the five regions.

## Discussion

### Principal Findings

Given the uncertainty and unavailability of data regarding health care parameters associated with COVID-19, we chose to implement a naïve model and fitting procedure in which the main intent was to produce forecasts of the expected health care use levels up to 4 weeks into the future. Although COVID-19 case data have been widely available, hospitalization data have been consistently sparse and not always timely. Therefore, alternative approaches such as naive models may prove to be more robust in addressing these challenges. Our evaluations show that using a simple model and available forecasts of cases, one can forecast future health care use levels with sufficient accuracy for operational planning. During the pandemic, these data were used across local health care systems to determine staffing, equipment, and contingency plans, enabling superior preparation for surges in cases. Additionally, transport logistics were informed by our forecasts, ensuring that communities had the necessary capabilities to move patients to higher levels of care.

We found that CHIVE varied in its ability to accurately forecast across space, time, and health care needs. First, CHIVE was more accurate and better calibrated at the state level than for the five regions of New Mexico. We may have obtained this result because although CHIVE forecasts the expected *needed* number of beds, ICU beds, and ventilators, decision-making on the ground of individual treatment and new incoming patient diversions based on capacity, staffing resources, etc, will impact these numbers. These individual decisions will have more of an impact on the regional numbers than overall state numbers. Second, at both geographic scales, the forecasts of inpatient beds had the smallest error, possibly because of fewer unaccounted-for downstream effects that could impact the number of ICU beds and ventilators. Third, the forecasting model was most accurate at the 1-week forecast horizon but improved its uncertainty coverage at the 2-week forecast horizon. Finally, CHIVE performed well in October and November, when confirmed COVID-19 cases and new hospitalizations were rising. This finding suggests this method is flexible for different phases of the epidemic. Overall, our results suggest that the conditions for which CHIVE is most suited include forecasting the number of hospital inpatient beds at higher geographical scales in the 2-week horizon.

Both a strength and limitation of our method is that it is dependent on the LANL COFFEE model. First, the resolution of the case forecasts limits the resolution of the hospitalization forecasts. At the state and regional levels, the model is not resolved enough for individual hospital planning in its current form; however, it can still provide intuition about how rising case numbers translate to stresses on capacity. Real-time awareness of where cases are rising and the features of specific hospitals (ie, rural vs urban) by public health professionals can provide synergistic information that can translate to an indication of where capacity may be stressed and needs to be reinforced. Second, the coverage, or prediction interval widths, were consistently too small. Because the distribution of case forecasts was used as input, this may be a reflection of overconfident intervals of the COFFEE model. Finally, both COFFEE and CHIVE are agnostic to on-the-ground public health actions. For example, the Governor of New Mexico reimposed a strict lockdown on November 13, 2020, to curtail rising case numbers [39]. The forecast for November 25 (Figure 4) overpredicted the number of inpatient beds, ICU beds, and ventilators. This may be because the effects of the stay-at-home order had yet not been observed in the data, so neither COFFEE nor the postprocessing step of CHIVE anticipated the reduction in new cases at the start of December.

Separate from COFFEE, a limitation of our method is that new hospitalizations are assumed to be a fraction of newly confirmed cases; meanwhile, data have shown that there is a median of 6 days between symptom onset and hospitalization and of 3 days between symptom onset and administration of a SARS-CoV-2 test [36]. Future iterations of the model could consider identifying the correct lag. However, we assume that lag is also a dynamic parameter. Finally, ALOS and other average values used are poor representations of the underlying distributions of hospital stays, which are known to have very long tails [40].

Nonetheless, we believe that given the availability of COFFEE forecasts for many geographic regions, this simple method could be used as a situational awareness tool for many health care departments across the nation (and even worldwide), who would only need to have their locale’s health care occupancy data available to supplement the forecasts of confirmed cases from COFFEE. For quantities that prove to be well calibrated, individual hospitals can use prediction intervals rather than point forecasts for their own needs. For example, if a hospital consistently sees their own caseloads around the lower 5% prediction interval, they can use this estimate for their individual needs. Alternatively, individual hospitals can use prediction intervals to determine their own risk avoidance by balancing the cost of unused beds versus going over capacity. In addition, this type of model could be used in nonpandemic settings where forecasts of disease burden take place, such as seasonal influenza.

The COVID-19 pandemic has highlighted the need for continued development of health care use forecasting. Seasonal hospitalization rates of influenza-like illnesses will be altered for years to come, compromising previous methodology that relied on historical time series to predict seasonal demand. Short-term forecasting may help state health departments and hospitals gain key situational awareness about what is expected in the near future. In addition, forecasting at a finer resolution, such as regions, can provide the opportunity for coordination of necessary resources. These modeling techniques may also prove helpful in addressing emergent needs in special and diverse populations that may otherwise go unmet and recognized. We see a strong and urgent need for continued collaboration between infectious diseases modelers, public health officials, and hospital managers. Although modelers can provide an outlook on transmission activity in the general population and translate transmission forecasts to incoming numbers and resources needed, hospital operations subject matter experts are best able to understand the limits of hospital capacities and resources, while public health officials can aid policy.

### Conclusions

Although there is uncertainty in our forecasts, the proposed methodology is intended to provide estimates to decision makers and public health officials regarding the potential need of health care resources resulting from a burgeoning pandemic. Specifically, the results of this study can help research groups, departments of health, and ministries of health estimate future health care needs and support decisions regarding resource planning and allocation to ultimately reduce negative health care outcomes and save lives.

## Appendix

Multimedia Appendix 1 Supplemental figures and tables.

## Abbreviations

ALOS: average length of stay
CDC: US Centers for Disease Control and Prevention
CHIVE: COVID-19 Hospitalization, Intensive Care, and Ventilator Estimator
COFFEE: COVID-19 Forecasts Using Fast Evaluations and Estimation
CSSE: Center of Systems Science and Engineering
DOE: Department of Energy
DHR: daily hospitalization rate
ICU: intensive care unit
JHU: Johns Hopkins University
LANL: Los Alamos National Laboratory
MAE: mean absolute error
NMDOH: New Mexico Department of Health
WAPE: weighted absolute percentage error
