# Supplementary material for: How New Mexico Leveraged a COVID-19 Case Forecasting Model to Preemptively Address the Health Care Needs of the State: Quantitative Analysis
Source: JMIR Public Health Surveill. 2021 Jun 9;7(6):e27888. doi: 10.2196/27888 (PMC8191729; doi:10.2196/27888)
Supplement: Multimedia Appendix 1 [file publichealth_v7i6e27888_app1.docx]

**Figure S1: New COVID-19 cases in New Mexico and regional level in-use healthcare resources from August through December 16^th^, 2020.** The top graph in each panel depicts the state-wide numbers, while the heatmaps below stratify the proportion of new cases (A), in-use hospital beds (B), occupied ICUs (C) and in-use ventilators (D) by region. The grey box denotes the period of forecasts for performance evaluation.

**Table S1: Weighted absolute percentage errors (WAPE) of the state-level forecasts using one to four weeks of back-fitting baseline forecasts, calculated across all healthcare resource categories.**

| **Back-fitting window (weeks)** | **Forecast Horizon – weeks ahead** | | | |
| --- | --- | --- | --- | --- |
|  | 1 | **2** | **3** | **4** |
| 1 | 0.170 | 0.246 | 0.315 | 0.345 |
| 2 | 0.199 | 0.277 | 0.339 | 0.362 |
| 3 | 0.218 | 0.297 | 0.355 | 0.369 |
| 4 | 0.221 | 0.306 | 0.366 | 0.372 |

**Fig S3:** **Coverage plot for forecasts made between September 1 and December 9, 2020 at the one- to four-week ahead horizons across the five New Mexico regions.** Colored lines are labeled by their nominal coverage, while the position on the y-axis indicates its empirical coverage. If a forecast is well-calibrated, the empirical coverage should fall along the y-axis at its corresponding nominal coverage.
